# The extended 4-year follow-up results of the ELOQUENT-2 trial

**DOI:** 10.18632/oncotarget.26527

**Published:** 2019-01-04

**Authors:** Maria Gavriatopoulou, Evangelos Terpos, Meletios Athanasios Dimopoulos

**Affiliations:** Department of Therapeutics, Alexandra General Hospital, National and Kapodistrian University of Athens, School of Medicine, Athens, Greece

**Keywords:** multiple myeloma, relapsed/refractory, elotuzumab, lenalidomide, extended PFS

Multiple myeloma is the second most common hematologic malignancy accounting for approximately 18% of all hematologic malignancies in the United States in 2016 [1]. Although the introduction of proteasome inhibitors and immunomodulatory agents in the treatment landscape of the disease has significantly improved the patients outcomes and has extended overall survival, multiple myeloma still remains incurable and all patients will eventually relapse. Therefore, the need for novel and effective therapeutic strategies is more than imperative. Under this prism, agents with novel mechanisms of action are combined with the standard regimens in order to overcome the drug resistance phenomenon and provide deep and durable responses also reflecting an advantage in overall survival. Elotuzumab is a humanized IgG1 monoclonal antibody targeting the signaling lymphocytic activation molecule F7 (SLAMF7), which is expressed on myeloma cells and natural killer cells but not on normal tissues [2]. Elotuzumab has a dual mechanism of action, by causing directly myeloma cell death through activation of natural killer cells or via antibody-dependent cellular cytotoxicity [2, 3]. It was demonstrated that the combination of elotuzumab with lenalidomide and dexamethasone (EloRd) further enhanced the activation of natural killer cells and subsequent myeloma cell death [4]. This triplet combination was evaluated in the randomized phase 3 ELOQUENT-2 trial compared to lenalidomide-dexamethasone (Rd) in patients with relapsed/refractory multiple myeloma (RRMM) who had received 1 to 3 prior lines of therapy and had documented disease progression after their most recent therapy [5]. The primary endpoints were progression free survival (PFS) and overall response rate (ORR). Overall survival (OS) was a secondary endpoint, while other exploratory endpoints included duration of response and safety. In previous analyses of the study at 2 and 3 years follow up, EloRd reduced the relative risk of disease progression or death by 30% and 27% respectively [5,6]. Among patients receiving the triplet combination the PFS rates demonstrated sustained relative improvements of 52% and 44% at 2 and 3 years respectively [5,6]. It was also demonstrated that the greatest PFS benefit was in favor of patients with a time from diagnosis longer or equal to the median of 3.5 years and especially for those with 1 prior line of therapy [5, 6]. These data probably indicate that slow progressors may particularly benefit from the addition of elotuzumab to Rd. Based on the survival benefit observed with elotuzumab at 2 and 3 years, an extended 4-year follow-up analysis was performed in order to evaluate the durability of response, including long term PFS and safety results. The median follow-up for patients without a PFS event was 46 months, while the median number of completed cycles was 19 for the EloRd arm and 14 for the Rd arm. Almost twice as many patients remained on treatment with EloRd (17%) compared to (9%) for Rd. The main reason for treatment discontinuation was disease progression, which was equally distributed among the 2 groups (54%), while the extended safety data were similar between the treatment arms. A relative improvement of 50% was observed in the 4-year PFS rate among the patients who received EloRd in comparison with Rd (21% *vs* 14%), while the PFS hazard ratio at the 4-year follow-up was 0.71 (95% CI, 0.59-0.86; *P* = .0004), which indicates a 29% reduction in the risk of disease progression or death in favor of EloRd. For patients achieving very good partial response or better a 35% reduction in the risk of progression or death was demonstrated in the EloRd arm (HR, 0.65; 95% CI, 0.46-0.94; *P* = .0208). PFS benefit in favor of EloRd was consistent across most patient subgroups, including patients above the age of 75 and patients with high-risk cytogenetics. Most importantly, relative risk reductions for progression or death of 36% (HR, 0.64; 95% CI, 0.43-0.97; *P* = .0331) and 23% (HR, 0.77; 95% CI, 0.62-0.95; *P* = .0159) were observed in favor of the triplet in patients with both high-risk and standard-risk disease, respectively. Furthermore, high-risk patients treated with EloRd had a median PFS that was 2 times longer than that of patients who received Rd (15 months with EloRd [95% CI, 9.3-21.2 months] *vs* 7 months with Ld [95% CI, 5.7-12.0 months]; HR, 0.64; 95% CI, 0.43-0.97; *P* = .0331). The ORR was 79% with EloRd and 66% with Rd. The duration-of-response benefit was maintained over time (HR, 0.77; 95% CI, 0.62-0.95; *P* = .0176) with a median duration of response of 21 months (95% CI, 18-26 months) with EloRd *versus* 17 months (95% CI, 15-19 months) with Rd. The early separation of OS curves previously reported was maintained over time in favor of EloRd with 4-year OS rates of 50% *versus* 43% for Rd (HR, 0.78; 95% CI, 0.63-0.96). The median OS was 48 months in the ELoRd arm and 40 months in the Rd arm. Furthermore, an indirect comparison based on data from other randomized phase 3 clinical trials of RRMM patients was performed in order to describe time-specific effects on the relative PFS of several triplet regimens with elotuzumab (EloRd), carfilzomib (KRd), ixazomib (IRd), and daratumumab (DRd) compared to Rd alone aiming to provide further insight of these regimens in the RRMM setting. This relative PFS analysis supported further the sustained efficacy of EloRd which was observed in the ELOQUENT-2 trial [7]. This study is of extreme importance due to the longest follow-up of any other monoclonal antibody in the RRMM setting. Furthermore, ELOQUENT-2 is the only randomized trial in this setting in which PFS data have been centrally assessed continuously after the median PFS goal was reached, while the early and persistent separation of the PFS and OS Kaplan Meier curves demonstrates the durable long-term clinical benefit provided by EloRd. Very recently, the role of elotuzumab with another IMiD compound-pomalidomide was reported for patients both refractory to lenalidomide and a proteasome inhibitor. These data indicated that the risk of progression or death was significantly lower for the patients who received the triplet combination compared to pomalidomide-dexamethasone [8]. The availability of novel treatment strategies has increased the complexity in choosing the most appropriate option in the RRMM setting. The above mentioned data strongly indicate that elotuzumab combined with lenalidomide and more recently with pomalidomide and dexamethasone are important treatment options with well-established safety and tolerability profile and supports the incorporation of these regimens as effective treatment options for the management of patients with RRMM.
